# Use of Humanized Mouse Models to Investigate the Roles of Purinergic Signaling in Inflammation and Immunity

**DOI:** 10.3389/fphar.2020.596357

**Published:** 2020-10-02

**Authors:** Ronald Sluyter, Debbie Watson

**Affiliations:** ^1^Illawarra Health and Medical Research Institute, Wollongong, NSW, Australia; ^2^Molecular Horizons and School of Chemistry and Molecular Bioscience, University of Wollongong, Wollongong, NSW, Australia

**Keywords:** adenosine A_2A_ receptor, CD39, CD73, graft-versus-host disease (GVHD), NSG mouse, P2X7 receptor, peripheral blood mononuclear cell (PBMC), xenogeneic mouse model

## Introduction

Purinergic signaling comprises a network of extracellular nucleosides and nucleotides, cell surface adenosine (P1) and nucleotide (P2) receptors, and ecto-enzymes that together participate in cell-to-cell communication (Giuliani et al., 2019). This network plays key roles in many physiological processes (Burnstock, 2012) including inflammation and immunity, as recently illustrated by members of the Italian Purine Club (Adinolfi et al., 2018; Di Virgilio et al., 2018; Magni et al., 2018; Antonioli et al., 2019) and others (Linden et al., 2019). Much of this understanding has been obtained from studies of cells expressing endogenous or recombinant purinergic molecules, rodent models of health and disease, and human tissue samples (Burnstock, 2012). Humanized mice provide a complementary approach to investigate purinergic signaling in inflammation and immunity and are valuable tools to translate findings from mice to humans. However, the use of humanized mice in this context is only in its infancy. In this opinion article, we will briefly provide a description of humanized mice. Then, using recent studies from our groups, we illustrate how a humanized mouse model has been used to advance our understanding of purinergic signaling in the inflammatory immune disorder, graft-*versus*-host disease (GVHD). Finally, directions for the future use of humanized mouse models to investigate purinergic signaling in inflammation and immunity and other systems will be briefly outlined.

## Humanized Mice

Humanized mice can be classified into two groups. The first involves the expression of specific human gene products within mice including cases in which a given mouse gene is replaced by the human ortholog (Stripecke et al., 2020). Examples of mice incorporating transgenes of human purinergic molecules include the overexpression of human CD39 (*ENTPD1*) (Dwyer et al., 2004), as well as the substitution of the mouse gene with the corresponding human gene for the adenosine A_3_ receptor (*ADORA3*) (Yamano et al., 2005), P2X7 receptor (*P2RX7*) (Metzger et al., 2017a) or a Gln460Arg P2X7 receptor variant (Metzger et al., 2017b). The second group of humanized mice, so called xenogeneic mouse models, involves the transfer of human cells into mice, which are typically immunodeficient (Stripecke et al., 2020). It is this group which forms the focus of the remaining article.

Humanized mice resulting from the engraftment of human cells have been important pre-clinical tools for three decades (Shultz et al., 2019). As such, there are a large number of humanized mouse models including those of relevance to inflammation and immunity, in which immunodeficient mice are engrafted with human peripheral blood mononuclear cells (PBMCs), hematopoietic cells or tissues to form functional human immune systems (Shultz et al., 2019). A brief history of the development of humanized mice, including a list of the current mouse platforms available and potential sources of human tissue, is provided elsewhere (Shultz et al., 2019).

The humanized mouse model most commonly used to investigate purinergic signaling in inflammation and immunity involves the injection of human PBMCs into non-irradiated NOD.Cg-*Prkdc^scid^IL2rg^tm1Wjl^* (NSG) mice (Hu-PBMC-NSG mice) (Geraghty et al., 2017), a model established by King et al. (2008). NSG mice readily engraft human cells due to naturally occurring and engineered mutations resulting in: impaired development of T and B cells and natural killer cells, preventing immune-mediated rejection of human cells; and enhanced mouse SIRPα-human CD47 interactions, promoting engraftment of human hematopoietic cells (Shultz et al., 2019). NODShi.Cg-*Prkdc^scid^IL2rg^tm1Sug^* (NOG) mice are similar to NSG mice except they encode a truncated, rather than a null, form of the IL-2 receptor *γ*-chain and can also engraft human PBMCs (Shultz et al., 2019). Thus, studies of NOG mice engrafted with human PBMCs provide supplementary information when seeking to understand immune mechanisms in Hu-PBMC-NSG mice. Studies of humanized NOG mice in relation to purinergic signaling are yet to be reported.

**Figure 1:**
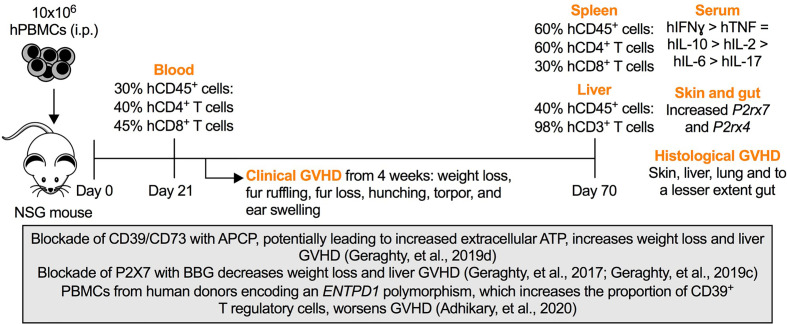
Purinergic signaling in a humanized mouse model of graft-*versus*-host disease (GVHD). Intraperitoneal (i.p.) injection of 10 × 10^6^ human (h) peripheral blood mononuclear cells (PBMCs) into NOD.Cg-*Prkdc^scid^IL2rg^tm1Wjl^* (NSG) mice (Day 0) results in the engraftment of human (h) CD45^+^ leukocytes predominately hCD4^+^ and hCD8^+^ T cells as early as Day 21. The percentages of hCD45^+^ leukocytes, hCD3^+^ T cells and hCD4^+^ or hCD8^+^ T cells represent the average percentages of these cells among total CD45^+^ leukocytes, hCD45^+^ leukocytes and hCD3^+^ T cells, respectively, typically observed in this model (Cuthbertson et al., 2020). From Week 4, mice display signs of clinical GVHD (as indicated) corresponding with the production of circulating human interferon-*γ* (hIFN (*γ*), tumor necrosis factor (hTNF) and interleukins (hIL) (as indicated) (Geraghty et al., 2017; Geraghty et al., 2019a; Geraghty et al., 2019d), increased murine *P2rx7* and *P2rx4* expression in GVHD tissues, and histological evidence of GVHD at endpoint (Day 70) (Cuthbertson et al., 2020). The box highlights studies of humanized mice in which roles for purinergic molecules were established as follows. Injection of the CD39/CD73 antagonist, *α,β*-methylene ATP (APCP), which potentially increases extracellular ATP, increases weight loss and liver GVHD (Geraghty et al., 2019d). Injection of the P2X7 antagonist, Brilliant Blue G (BBG), decreases weight loss and liver GVHD (Geraghty et al., 2017; Geraghty et al., 2019c). Injection of PBMCs from human donors encoding an *ENTPD1* polymorphism, which increases the proportion of CD39^+^ T regulatory cells, worsens GVHD (Adhikary et al., 2020).

A number of features need to be considered when studying purinergic signaling pathways in Hu-PBMC-NSG mice. First, despite readily engrafting human T cells, the engraftment of human B cells and myeloid cells in these mice is limited (King et al., 2008), presumably due to species-specific factors (Shultz et al., 2019). Second, these factors are likely to disrupt the engraftment of other human leukocyte subsets, such as the observed decline of human T regulatory cells in these mice over time (Hu et al., 2020). Third, NSG mice display defects in other immune pathways such as the complement pathway (Verma et al., 2017) limiting the scope of studying some inflammatory and immune processes. Fourth, disparities between murine MHC class I and II molecules and human T cell receptors may yield sub-optimal human immune responses (Lee et al., 2019). Fifth, NSG mice display higher rates of antibody clearance compared to other strains (Li et al., 2019) reducing the efficacy of functional monoclonal antibodies in this model. Finally, Hu-PBMC-NSG mice develop lethal GVHD from 4 weeks (King et al., 2009; Geraghty et al., 2019b), limiting long-term studies in these mice. This last feature however affords a valuable pre-clinical model of this disease, which we have utilized to investigate the role of purinergic signaling pathways in GVHD (**Figure 1**).

## Purinergic Signaling in GVHD in Humanized NSG Mice

Allogeneic hematopoietic stem cell transplantation (HSCT) is a curative therapy in people with malignant and other blood disorders (Copelan et al., 2019). However, GVHD, in which donor immune cells damage and destroy host tissues occurs in up to 30% of HSCT recipients, leading to severe morbidity and high rates of death (Zeiser and Blazar, 2017). GVHD typically occurs in the skin, intestines, liver and lungs, but can extend to the eyes, ovaries and brain (Zeiser and Blazar, 2017). As such, new and additional treatments are needed to further decrease the impact and incidence of GVHD in HSCT recipients.

Studies from allogeneic mouse models of GVHD, in which donor leukocytes from one mouse strain are transplanted into a second mouse strain, have revealed important roles for purinergic signaling pathways in GVHD development, identifying new potential therapeutic targets in preventing this disease in humans. Using small molecule antagonists/agonists and knockout mice of purinergic molecules, these studies have revealed that ATP is released at sites of inflammation and that P2X7 receptor activation on host antigen presenting cells contributes to the stimulation of donor effector T cells to promote GVHD progression (Wilhelm et al., 2010). Moreover, P2Y_2_ receptor activation on host cells contributes to this disease by directing monocytes to sites of inflammation and causing the apoptotic loss of intestinal cells (Klämbt et al., 2015). Conversely, adenosine A_2A_ receptor activation by CD73-generated adenosine limits GVHD progression (Lappas et al., 2010; Tsukamoto et al., 2012), an effect mediated in part by the expansion of donor T regulatory cells (Han et al., 2013). Collectively, these data suggest a working paradigm in which extracellular ATP activates P2 receptors to promote inflammation and GVHD, while extracellular adenosine activates adenosine receptors to limit inflammation and GVHD.

To determine if the above paradigm is relevant to human GVHD, our groups have investigated the roles of purinergic signaling in Hu-PBMC-NSG mice using small molecule antagonists/agonists of purinergic molecules and PBMCs from human donors encoding natural variants of the *P2RX7* and *ENTPD1* genes (**Figure 1**). Collectively, this data supports the role of extracellular ATP (Geraghty et al., 2019d) and the subsequent activation of the P2X7 receptor (Geraghty et al., 2017; Geraghty et al., 2019c) in promoting GVHD, most notably liver GVHD, in this humanized mouse model. This effect appeared to be due to activation of host P2X7 receptors, as PBMCs from human donors encoding either loss-of-function or gain-of-function *P2RX7* gene variants resulted in similar rates and severity of GVHD (Adhikary et al., 2019). In contrast, a role for CD73-derived adenosine and A_2a_ receptor activation in preventing GVHD in Hu-PBMC-NSG mice could not be established (Geraghty et al., 2019d). Use of the adenosine A_2a_ receptor agonist, CGS 21680, suggested a role for this receptor in preventing GVHD progression, but this result was confounded by this agonist increasing weight loss in Hu-PBMC-NSG mice (Geraghty et al., 2019a). Further complicating an immunosuppressive role for adenosine in this model, is our observation that engraftment of human PBMCs with a polymorphic variant of the *ENTPD1* gene, that results in increased CD39^+^ T regulatory cells, worsens GVHD (Adhikary et al., 2020). Finally, our studies have revealed increased expression of murine *P2rx7* and *P2rx4* in GVHD tissues from Hu-PBMC-NSG mice compared to those from non-engrafted NSG mice (Cuthbertson et al., 2020) and the presence of functional murine P2X7 receptors in NSG mice (Geraghty et al., 2017), whilst both human *P2RX7* and *ADORA2* are detected in Hu-PBMC-NSG mice (Geraghty et al., 2019d). Collectively, this data suggests Hu-PBMC-NSG mice provide a pre-clinical model of GVHD in which new therapeutics aimed at inhibiting P2X7 receptor activation can be tested, whilst the potential use of this model to test new therapeutics aimed at activating A_2A_ receptors remains to be established. Moreover, through the use of species-specific biologics (Koch-Nolte et al., 2019), Hu-PBMC-NSG mice afford new opportunities to delineate the role of donor (human) and host (murine) purinergic molecules in GVHD. One caveat in using Hu-PBMC-NSG mice to study purinergic signaling in GVHD is that the use of purinergic antagonists/agonists in these mice are typically less effective in modifying disease outcomes than in allogeneic mouse models of GVHD. This difference most likely reflects the greater disparity in MHC molecules between species than between mouse strains resulting in more severe forms of GVHD in Hu-PBMC-NSG mice compared to allogeneic mice.

## Conclusions and Future Directions

Due to the development of lethal GVHD in Hu-PBMC-NSG other studies of purinergic signaling in inflammatory and immune processes in these mice remain limited. Nevertheless, given these mice readily engraft human T cells, these mice present opportunities to study the role of purinergic molecules in human T cell activation, differentiation, migration and survival *in vivo* for up to 4 weeks prior to clinical GVHD development. Moreover, the above studies of purinergic signaling in GVHD in Hu-PBMC-NSG mice serve as a proof-of-concept to consider studying the roles of purinergic signaling in inflammatory and immune processes in other humanized mouse models. In this regard, recent advances, such as the expression of transgenes for human growth factors and use of human progenitor cells, have facilitated the engraftment of human T cells and other human leukocytes in the absence of GVHD (Stripecke et al., 2020). Other advances have assisted the study of human T cell responses *in vivo*. For example, expression of human MHC class I and II transgenes in NSG mice has facilitated the study of CD8^+^ and CD4^+^ T cell responses in graft-*versus*-leukemia immunity (Ehx et al., 2018) and colitis (Goettel et al., 2016), respectively, in Hu-PBMC-NSG mice. Thus, purinergic investigators seeking to employ humanized mice need to consider the purinergic pathway(s) and cell type(s) of interest in selecting the most appropriate humanized mouse model available, including the development of new humanized mouse models to address aims.

In wanting to employ humanized mice, investigators also need to consider the ethical implications and constraints of using animals and human tissues, including the generation of human–mice chimeras and the source of human cells (Devolder et al., 2020), with some humanized mouse models requiring human fetal liver tissue (Shultz et al., 2019). Nevertheless, given the range of humanized mouse models emerging (Stripecke et al., 2020), humanized mice provide new and exciting opportunities for the study of purinergic signaling in inflammation and immunity, as well as in other physiological and pathophysiological settings. For example, von Willebrand factor mutant mice, which support human but not murine platelet-induced thrombosis, have been used to study the P2Y_12_ receptor antagonist clopidogrel *in vivo* (Magallon et al., 2011). Additionally, given the roles of purinergic signaling in cancer progression and metastasis, as highlighted by members of the Italian Purine Club (Di Virgilio and Adinolfi, 2017; Ferrari et al., 2017; Giuliani et al., 2018), human tumor xenograft models will support the future study of such pathways in this disease. Humanized mouse models also afford opportunities to develop personalized medicine relating to purinergic targets in disease, as illustrated by the use of human tumor xenografts (so called patient-derived xenograft or PDX models) in tailoring therapies for people with cancer (Shultz et al., 2019). Finally, investigators are directed to recent standardized reporting guidelines concerning the use of humanized mice aimed at enhancing rigor and reproducibility (Stripecke et al., 2020).

## Author Contributions

RS wrote the manuscript and prepared the figure. DW provided additional commentary and edited the manuscript and figure. All authors contributed to the article and approved the submitted version.

## Funding

RS and DW are supported by funds from Molecular Horizons, University of Wollongong (Wollongong, Australia). DW is supported by project grants from the Cancer Council NSW and the Faculty of Science, Medicine and Health, University of Wollongong.

## Conflict of Interest

The authors declare that the research was conducted in the absence of any commercial or financial relationships that could be construed as a potential conflict of interest.
